# Carrier dynamic identification enables wavelength and intensity sensitivity in perovskite photodetectors

**DOI:** 10.1038/s41377-024-01636-6

**Published:** 2024-09-29

**Authors:** Liangliang Min, Yicheng Zhou, Haoxuan Sun, Linqi Guo, Meng Wang, Fengren Cao, Wei Tian, Liang Li

**Affiliations:** 1 School of Physical Science and Technology, Jiangsu Key Laboratory of Frontier Material Physics and Devices, Center for Energy Conversion Materials & Physics (CECMP), Soochow University, Suzhou, China; 2 College of Physical Science and Technology & Microelectronics Industry Research Institute, Yangzhou University, Yangzhou, China

**Keywords:** Optical sensors, Optoelectronic devices and components, Imaging and sensing

## Abstract

Deciphering the composite information within a light field through a single photodetector, without optical and mechanical structures, is challenging. The difficulty lies in extracting multi-dimensional optical information from a single dimension of photocurrent. Emerging photodetectors based on information reconstruction have potential, yet they only extract information contained in the photoresponse current amplitude (responsivity matrix), neglecting the hidden information in response edges driven by carrier dynamics. Herein, by adjusting the thickness of the absorption layer and the interface electric field strength in the perovskite photodiode, we extend the transport and relaxation time of carriers excited by photons of different wavelengths, maximizing the spectrum richness of the edge waveform in the light-dark transition process. For the first time, without the need for extra optical and electrical components, the reconstruction of two-dimensional information of light intensity and wavelength has been achieved. With the integration of machine learning algorithms into waveform data analysis, a wide operation spectrum range of 350–750 nm is available with a 100% accuracy rate. The restoration error has been lowered to less than 0.1% for light intensity. This work offers valuable insights for advancing perovskite applications in areas such as wavelength identification and spectrum imaging.

## Introduction

**Fig. 1:**
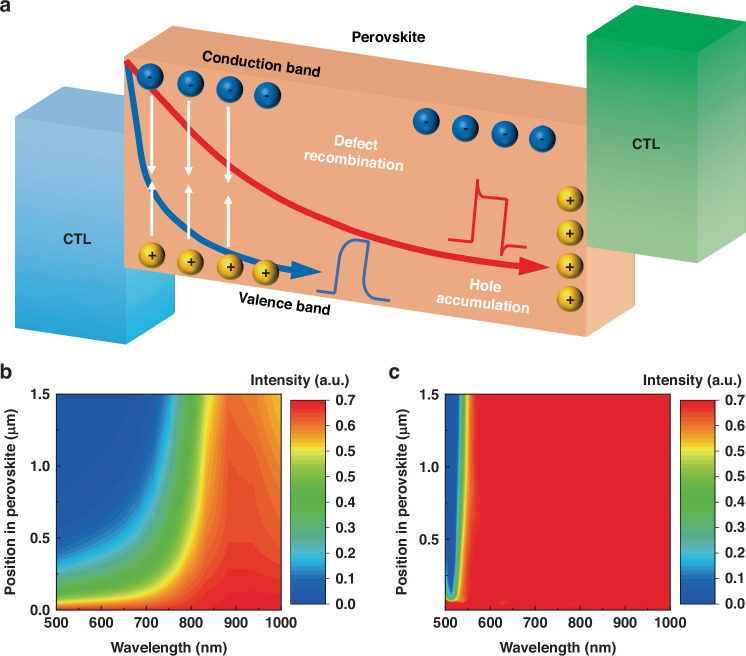
Device design principle. **a** Schematic diagram of photogenerated carrier transport routes and photocurrent waveforms under long wavelength and short wavelength irradiation. The optical field distributions in **b** 3D MAPbI_3_ and **c** 2D (PEA)_2_PbI_4_ under different wavelengths analyzed by FDTD simulation

Traditional photodetectors only have the ability to roughly calibrate the intensity of the source light^1,2^. In recent years, by adding optical components such as filters^3–5^, polarizers^6,7^, metasurface structures^8–10^ to traditional photodetectors, or through in-situ voltage modulation^11–13^ and systematized measures such as multi-photodetector integration^14–16^, photodetectors have been endowed with sensitivity to different dimensional information such as wavelength, polarization, and phase. Subsequently, by employing information reconstruction techniques, these photodetectors have realized the capability for multi-dimensional detection and comprehensive analysis of incident light^17^. However, these strategies don’t make full use of the detected information. Taking the wavelength-sensitive photodetector as an example, whether employing optical dispersion or in situ voltage modulation, only the spectral richness information implied in the photoresponse current amplitude (responsivity matrix) is extracted^18–21^, and the information contained in the edge waveform during the light-dark transition is neglected. As shown in Fig. 1a, with the increase of photon energy, the transport and relaxation process of carriers requires a longer transport distance, which will increase the equivalent transmission impedance of the device. Thus, the waveform of the response edge can be regulated. Theoretically, this carrier transport process characteristic that changes with wavelength can serve as an indicator of the energy of incident light. It is worth noting that although most photodetectors provide current versus time curves under different wavelengths^22–24^, they generally display consistent pattern (either all overshoot or flatness), rarely reflecting the complete evolutionary process mentioned above. Thus, it becomes crucial to pinpoint a promising material, wherein the device’s impedance is modulated under diverse photon excitation conditions, aiming to optimize the spectral richness of the device within the available wavelength spectrum.

Due to the flexibility in component adjustment, metal halide perovskites is promising^25^. It not only has a continuously adjustable band structure, its light absorption coefficient^26^, the strength and distribution of the built-in electric field in the body phase, can all be continuously adjusted by a simple solution method^27,28^. In addition, as shown in Figure S1 (Supporting Information), in the time-resolved photoluminescence (TRPL) spectra of two-dimensional (2D) perovskites under excitation of varying wavelengths and light intensities, a pronounced wavelength and intensity-dependent carrier lifetime can be observed. Consequently, 2D perovskites have been selected as the design exemplar.

In this work, we propose a brand-new concept to distinguish the wavelength and intensity of monochromatic light solely based on the photocurrent waveform without any supplementary systems. The composition distribution of the absorption layer, specifically (PEA)_2_MA_3_Pb_4_I_13_ (where MA represents methylammonium and PEA denotes phenethylammonium), is strategically adjusted along the direction of light propagation. This manipulation controls the sites where carriers, excited by photons of different energies, are generated. As a result, the device exhibits a differentiated transmission impedance when subjected to light injection of varying wavelengths. In addition, the modulation of the charge transport layer (CTL) against the direction of the 2D perovskite’s inherent electric field induces diverse temporal variations in the photocurrent waveform under different wavelengths within the 350-750 nm range. The integration of machine learning algorithms into waveform data analysis has led to a remarkable 100% accuracy rate in identifying the wavelength of unknown incident light, and a reduction in the restoration error for light intensity to less than 0.1%.

## Results

### Design principle

As illustrated in Fig. 1a, the absorption of short-wavelength light and the generation of carriers are mainly confined to the bottom surface. While a large number of defects are concentrated at the surface, the carrier will be trapped by these defects during the transport process, resulting in a slowly increasing photocurrent curve waveform. The absorption of long-wavelength light is mainly concentrated in the top bulk of the perovskite. Due to the cavity effect, these lights will generate more carriers with less recombination loss^19^. The thickness of the perovskite film was initially considered for different wavelength light absorption at different depths. The finite-difference time-domain (FDTD) simulation was conducted to analyze the optical field distributions in 3D MAPbI_3_ and 2D (PEA)_2_PbI_4_ under different wavelengths, as shown in Fig. 1b, c, respectively. The light penetration depth of (PEA)_2_PbI_4_ perovskite before 530 nm wavelength is below 100 nm, while the light penetration depth of MAPbI_3_ perovskite at its absorption edge can reach about 1 μm. Considering that the absorption edge of three-dimensional-like (3D-like) perovskite is slightly smaller than the corresponding 3D perovskite due to the influence of quantum confinement effect^29^, it is preliminarily believed that the thickness of the MA-based 2D perovskite should be slightly less than 1 μm to achieve full absorption of all available absorption spectrum. The inverted structured devices can generate fast photoresponse under different wavelength light, and obtain horizontal and vertical photo-dark current curves without any spike or ramp. Due to the special nucleation and crystallization process, the 2D perovskite will form a gradient phase component distribution from large n-phase to small n-phase from top to bottom^30^. The resulting gradient bandgap determines that it is suitable for the preparation of inverted structured devices for fast carrier transmission. In short, by manipulating the light absorption, charge transport and collection characteristics in the perovskite and CTLs, it is feasible to distinguish wavelength by the photocurrent waveform. The penetration depth of short-wavelength light is much shallower than that of the long-wavelength light (Fig. 1b, c).

### 2D perovskite film characterization

**Fig. 2:**
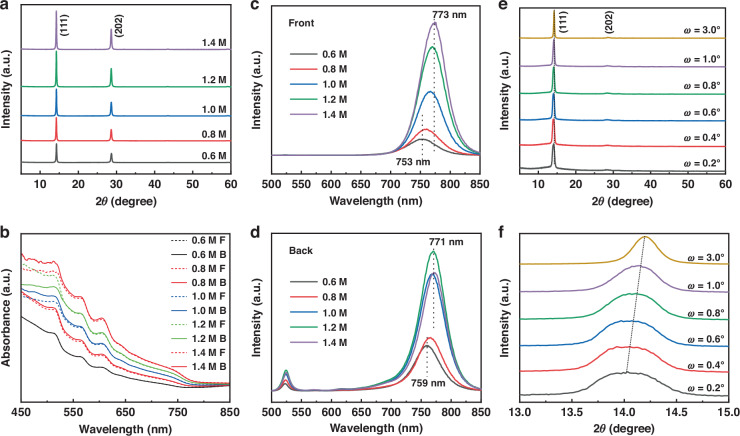
Characterization of crystallinity and phase distribution of perovskite. **a** X-ray diffraction (XRD), **b** UV-vis absorption spectrum from the back side (solid line) and the front side (dash line) and Photoluminescence (PL) spectrum from **c** the back side and **d** the front side of 2D perovskite films with different precursor solution. **e** Glancing-angle XRD (GIXRD) of 1.2 M 2D perovskite film and **f** its enlargement near the plane of (111)

2D perovskite films of varying thicknesses were fabricated using the hot casting method with precursor solutions of 0.6, 0.8, 1.0, 1.2, and 1.4 M concentration. The corresponding top-view and cross-sectional scanning electron microscopy (SEM) images are depicted in Figure S2 (Supporting Information). Notably, all films exhibit dense and uniform surfaces, with thicknesses ranging from 357 to 431, 594, 837, and 1025 nm, respectively. X-ray diffraction (XRD) analysis was conducted to examine the crystallinity and crystal orientation of the 2D perovskite films. As illustrated in Fig. 2a, all films exhibit distinctive diffraction peaks at 14.2° and 28.5°, attributed to the (111) and (202) crystal planes, respectively^31^. The absence of other peaks suggests that these films grow perpendicular to the substrate, facilitating efficient charge transport between different n-phase perovskite layers^32^. Moreover, the peak intensity gradually increases with increasing perovskite film thickness. UV-vis absorption spectrum was employed to assess the phase distribution of the 2D perovskite films along the vertical direction. As shown in Fig. 2b, the absorption peaks appear at 514, 566, 606, 644, and 750 nm, corresponding to *n* = 1, 2, 3, 4, and 3D-like phase components, respectively^33^. Notably, as the perovskite thickness increases, the absorption intensity also rises. Additionally, stronger absorption from the back side (glass side) compared to the front side (upper surface of perovskite) is observed, particularly for wavelengths corresponding to *n* ≤ 4 components, indicating a concentration of 2D perovskites primarily on the side near the substrate. Photoluminescence (PL) spectrum analysis is also an effective means to characterize the phase distribution. Since the excitation light with a wavelength of 470 nm and an incident angle of 45° has a limited penetration depth (less than 100 nm), the information in the PL spectrum is limited to the incident surface. Excitation peaks corresponding to 3D-like components near 750 nm are evident on both front and back sides (Fig. 2c, d). On the back side, distinct excitation peaks corresponding to n = 1, 2, and 3 phase components are observed at 518, 572, and 618 nm, respectively, whereas no such peaks are observed on the front side, indicating that all perovskite films are composed of different phase components, and more 2D perovskite phase components with small n values are concentrated at the back side of the film^34^. To delineate the depth profile of the these 2D perovskite films, glancing-angle XRD (GIXRD) analysis was performed^35^. The 1.2 M perovskite film was selected as a representative in Fig. 2e, f. It is evident that with an increase in glancing-angle, the (111) peak position shifts gradually to higher 2*θ* angles. The peak position of (110) crystal plane for MAPbI_3_ is around 14°^36^. With the insertion of large 2D cation ammonium salt, the peak position will have a displacement toward higher 2*θ* angles. The concentration of PEA exerts a significant influence on the lattice parameters of the 2D perovskite, encompassing both the magnitudes and orientations of the lattice vectors^31,37^. Consequently, any alterations in the phase components along the vertical direction will induce modifications in the interplanar distances of the (111) crystallographic plane. This observation suggests that there is a gradual reduced crystal lattice from the upper surface to bottom of the film, consistent with the distribution trend of phase components from 3D (top) to 2D (bottom). The GIXRD patterns of other four thickness films are shown in Figure S3 (Supporting Information), which has the same trend as the 1.2 M film. To further substantiate the gradient phase distribution, cross-sectional Kelvin probe force microscopy (KPFM) was employed to ascertain the contact potential difference (CPD) in the vertical direction (Figure S4, Supporting Information). The CPD value, which is intrinsically linked to the Fermi level, exhibits a gradual increase from the top surface towards the bottom, with a maximum discrepancy of approximately 100 meV. This observation unequivocally validates the gradient phase distribution from top to bottom.

### Modulation of Perovskite and CTLs

**Fig. 3:**
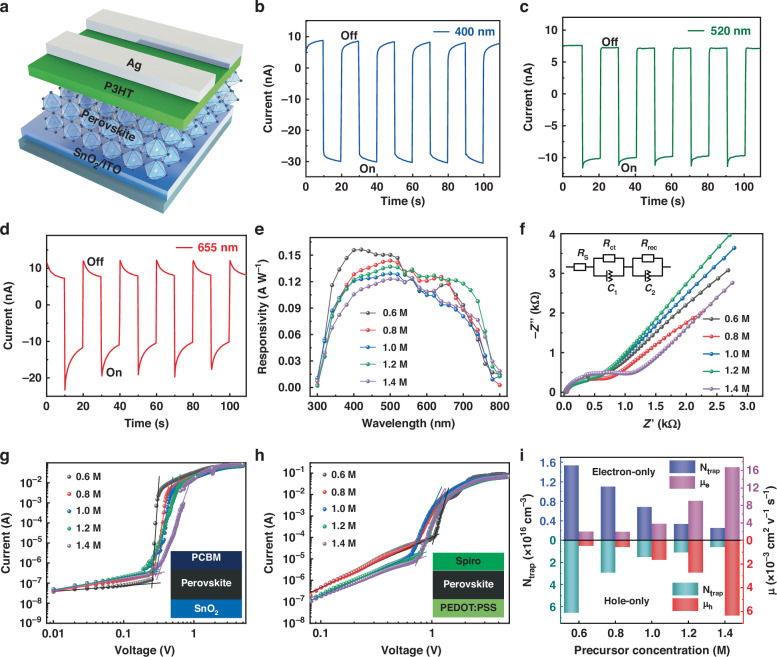
Device response and carrier dynamic in perovskite film. **a** The device architecture of the n-i-p self-powered photodetectors. The photocurrent curve waveforms under the excitation of **b** 400 nm, **c** 520 nm and **d** 655 nm. **e** The responsivity spectrum of the photodetectors with different perovskite thickness at 0 V bias. **f** The electrochemical impedance spectroscopy (EIS) of the photodetectors with different perovskite thickness. Space-charge-limited current (SCLC) of different perovskite thickness with **g** electron-only and **h** hole-only devices. **i** Calculated trap density and carrier mobility from **g** and **h**

For different photocurrent waveforms under different lights, the normal structured device was constructed. The device architecture of our n-i-p self-powered photodetectors is illustrated in Fig. 3a, wherein tin dioxide (SnO_2_) and poly (3-hexylthiophene-2,5-diyl) (P3HT) act as electron transport layer (ETL) and hole transport layer (HTL), respectively. To elucidate the factors influencing the waveform, the thickness parameter of each layer, from bottom to top, was explored. Initially, commercially purchased SnO_2_ colloidal dispersion with a weight ratio of 15% was diluted with various volumes of water to achieve different thicknesses of ETLs at the same spin-coating speed (Figure S5, Supporting Information). Devices with varied thicknesses of ETLs exhibited consistent waveforms under different wavelength lights, with the ETL thickness solely affecting the photocurrent magnitude—the thicker the ETL layer, the higher the photocurrent. In pursuit of optimal photoelectric performance, undiluted SnO_2_ stock solution was chosen as the precursor solution for ETL preparation. Compared with the ETL, the thickness of HTL notably influences the waveform. By selecting P3HT with low hole mobility as the HTL and adjusting its thickness, holes cannot be efficiently extracted, leading to hole accumulation at the interface, resulting in an ‘h’-shaped photocurrent waveform. As shown in Figure S6 (Supporting Information), when the HTL is too thin (5 mg mL^−1^), it does not hinder the carrier transport, and the photocurrent at any wavelength shows a fast response without any spike or ramp. While the HTL is too thick (≥15 mg mL^−1^), it will significantly hinder the carriers transport at any wavelength, manifesting a slow trapping process in the corresponding photocurrent waveform. When the HTL concentration is 10 mg mL^−1^, the photocurrent curves at different wavelengths exhibit distinct waveforms. Optical simulation in Fig. 1a suggests that for effective light absorption below 770 nm for the 3D-like phase, the perovskite layer’s thickness should exceed 500 nm. A correlation between the thickness of the perovskite layer and the penetration depth of light at different wavelengths is anticipated. As demonstrated Figure S7a and S7b (Supporting Information), when the thickness of perovskite is less than 500 nm, all wavelength photocurrent curves exhibit sharp peak waveforms. At a thickness of 594 nm, the photocurrent curve for red and green light remains sharply peaked, whereas that for blue light exhibits a horizontal waveform (Figure S7c) (Supporting Information). With the perovskite layer thickness further increasing to 837 nm, three distinct photocurrent waveforms emerge in three different optical bands (Fig. 3b–d), enabling simple RGB color discrimination. However, when the perovskite layer exceeds 1025 nm, carriers generated under all wavelength lights will be trapped, resulting in a slowly increasing photocurrent curve waveform.

### Optoelectronic and electrochemical properties

The device structure ITO/SnO_2_/1.2 M Perovskite/10 mg mL^−1^ P3HT/Ag was identified as the optimal configuration for color discrimination. To elucidate the factors contributing to color discrimination under this setting, several photoelectric and electrochemical characterizations were conducted. Responsivity (*R*), a critical parameter for assessing the sensitivity of a photodetector, measures the efficiency of the device in responding to optical signals. It is defined as the ratio of photocurrent to incident light intensity:

$$R=\frac{{I}_{{\rm{ph}}}-{I}_{{\rm{d}}}}{{P}_{{\rm{light}}}\cdot S}$$

where *I*_ph_, *I*_d_, *P*_light_, and *S* represent the photocurrent, dark current, light intensity, and device active area, respectively. Figure 3e shows the responsivity spectrum of the photodetectors with varying perovskite thickness at 0 V bias. As the perovskite thickness increases, there is a decline in responsivity for wavelengths below 500 nm, while responsivity tends to increase for wavelengths exceeding 600 nm. The increase of perovskite layer thickness enhances light absorption, leading to a gradual increase in photocurrent generated by longer wavelength light. However, photocurrent generated by shorter wavelength light must traverse a longer distance to reach the opposite electrode, resulting in reduced conversion efficiency from charge to current, hence, the attenuation of responsivity. When the perovskite layer becomes excessively thick, even with adequate light absorption, the transport and collection of carriers become increasingly challenging. Hence, at a concentration of 1.4 M, the overall responsivity diminishes entirely. Notably, despite the unfavorable thickness of perovskite and the energy level structure of the device for carrier transport, the device based on a 1.2 M perovskite precursor still exhibits a considerable responsivity of 0.137 A W^−1^. The electrochemical impedance spectroscopy (EIS) measurement was conducted to analyze the carrier transport and recombination behaviors (Fig. 3f). The fitted charge transport resistance (*R*_ct_) and charge recombination resistance (*R*_rec_) for all these devices are presented in Table S1 (Supporting Information). The *R*_ct_ first decreases to the minimum value for 1.2 M and then increases, while the *R*_rec_ exhibits the opposite trend, indicating a relatively faster carrier transport and reduced carrier recombination for 1.2 M perovskite precursor-based device. To thoroughly understand the impedance information of charge carriers generated under different monochromatic lights, we conducted EIS measurement under the condition that the device was irradiated with the same number of photons for each monochromatic light (Figure S8, Supporting Information). As the wavelength increases, the *R*_ct_ of the charge carriers gradually decreases, while the *R*_rec_ progressively increases, which is consistent with the design principle shown in Fig. 1a. The space-charge-limited current (SCLC) measurement is widely employed to assess trap density and carrier mobility in semiconductors. Electron-only and hole-only devices were fabricated with the following architectures: ITO/SnO_2_/Perovskite/PCBM/Ag and ITO/PEDOT:PSS/Perovskite/P3HT/Ag, as depicted in Fig. 3g and h, respectively. The trap density of perovskite film can be quantitatively determined with the following equation:

$${N}_{{\rm{t}}}=\frac{2{\varepsilon }_{0}{\varepsilon }_{{\rm{r}}}{V}_{{\rm{TFL}}}}{e{L}^{2}}$$

where *ε*_0_, *ε*_r_, *V*_TFL_, *e*, and *L* represent the vacuum permittivity, the relative dielectric constant of perovskite, the onset voltage of the trap-filled limit region, the elemental charge, and the thickness of the perovskite film, respectively. In the *J*-*V* curves, *J* is proportional to *V* in the ohmic region at low voltage, while *J* is proportional to *V*^2^ obeying Mott-Gurney law in the SCLC region at high voltage. The carrier mobility can be described by the following equation:

$$J=\frac{9\mu {\varepsilon }_{0}{\varepsilon }_{r}{V}^{2}}{8{L}^{3}}$$

where *μ* is the mobility. It is worth noting that the relative electrical permittivity of perovskite films with different thicknesses was measured by EIS, as shown in Figure S9 (Supporting Information)^38,39^. The relative electrical permittivity increases with the increase of thickness, and reaches the maximum at 1.2 M. Larger electrical permittivity can lead to lower exciton binding energy, thus provide more effective separation of electrons and holes, and facilitate the trapped electron to escape from deep level defect states, consistent with the EIS results. The calculated trap density and carrier mobility are shown in Fig. 3i. The trap density (hole and electron) decreases monotonically with the increase of perovskite thickness, while the carrier mobility (hole and electron) increases monotonically. The response time is a crucial metric for evaluating the performance of photodetectors under high-frequency conditions, defined as the time difference between 10% and 90% of the response amplitude. Owing to the wavelength sensitivity characteristic of the photodetector, the response time varies with incident wavelength. As shown in Figure S10 (Supporting Information), the response time of the device under different wavelengths are depicted. To ensure accurate determination of unknown light wavelengths and intensities, the response time of the device should be taken as the maximum value, which is approximately 7 µs under 450 nm. Such a short response time is sufficient to ensure the rapid identification of unknown monochromatic light.

### Characterization of competitive built-in electric field

**Fig. 4:**
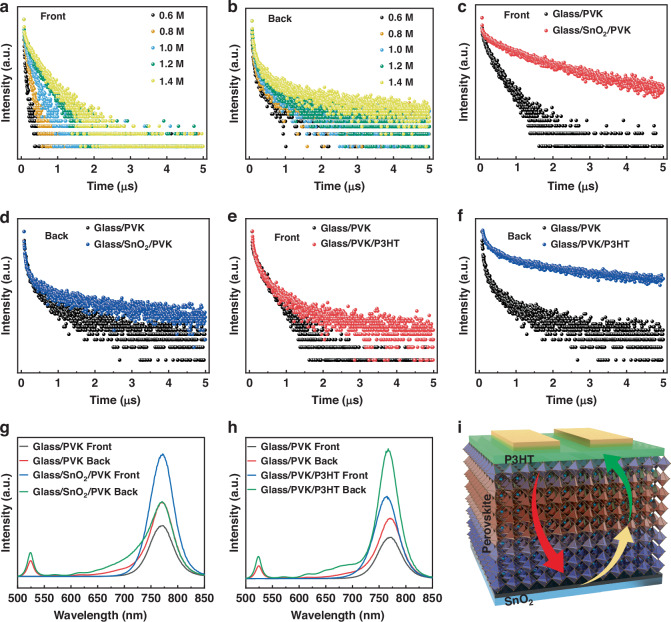
The built-in potential direction between the perovskite and carrier transport layers. The **a** front and **b** back TRPL curves of different thickness perovskite films. The **c** front and **d** back TRPL curves of the perovskite film with/without SnO_2_. The **e** front and **f** back TRPL curves of the perovskite film with/without P3HT. PL spectrum of the perovskite films with/without **g** SnO_2_, **h** P3HT. **i** The built-in field direction of the SnO_2_/perovskite/P3HT structure device is as follows: The yellow arrow represents the built-in field at the SnO_2_/perovskite interface, the green arrow signifies the built-in field at the perovskite/P3HT interface, and the red arrow illustrates the built-in field within the 2D perovskite

The monotonic change of trap density and carrier mobility is not enough to explain the change of curve waveform. TRPL and steady-state photoluminescence (PL) measurements were conducted to analyze the influence of electric fields at the junctions (interface between perovskite and charge transport layers, CTLs). Different thicknesses of perovskite films were directly deposited on glass substrates, and the TRPL curves for the front and back sides are depicted in Fig. 4a, b, respectively. By fitting the curves with the biexponential rate law, the fast, slow, and average recombination time constants are presented in Table S2 (Supporting Information). The average carrier lifetime (*τ*_ave_) increases with the thickened perovskite film for both front and back sides, while all the *τ*_ave_ of the back side is longer than that of the front one. The ordered phase distribution makes the perovskite film has built-in electric field. A large built-in field can make the carrier transfer faster, which is reflected in the reduction of carrier lifetime^40^. Therefore, the built-in field intensity on the upper surface of perovskite is stronger than that on the lower surface. Then 1.2 M perovskite precursor was spin coated on the glass/SnO_2_ substrate, it was found that the *τ*_ave_ on the front side increases sharply from 15.3 ns to 392.0 ns (Fig. 4c), while the *τ*_ave_ on the back side decreases from 34.6 ns to 15.6 ns compared with the glass substrate (Fig. 4d). The direction of the built-in field generated by SnO_2_/perovskite is against the direction of the built-in field of perovskite itself, and has a greater impact on the field from the upper surface. Similarly, glass/perovskite/P3HT sample was also tested in the same manner (Fig. 4e, f). The front *τ*_ave_ is almost unchanged, while P3HT increases the back *τ*_ave_ by about three times, indicating that P3HT has a significant influence on the built-in field on the lower surface of perovskite. Steady-state PL measurements on the front and back sides further corroborate the existence of the reverse field against the perovskite itself. The reverse field hinders the movement of the carriers for radiation recombination, thereby reducing the recombination loss during the transport process^40^. Deposition of CTLs results in an increasing trend in PL intensity for both front and back sides (Fig. 4g, h), providing further evidence that the field generated between the CTLs and perovskite opposes that of the perovskite itself. The direction of the built-in field for the entire device is depicted in Fig. 4i.

### Light wavelength and intensity identification

**Fig. 5:**
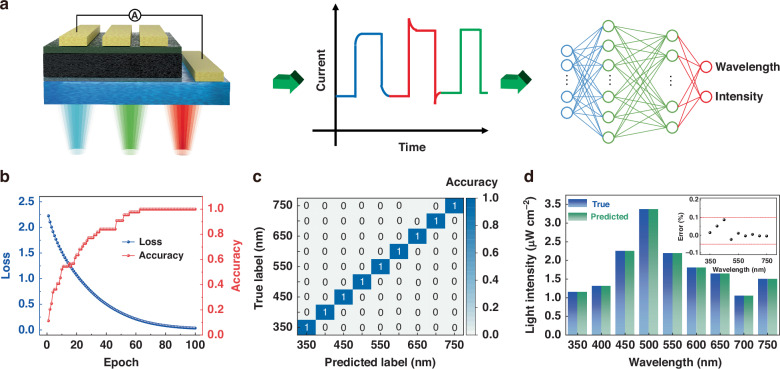
Deep learning for spectral reconstruction. **a** Schematic diagram of the wavelength and light intensity identification process. **b** Loss and accuracy of the neural network model during training. **c** Wavelength and **d** light intensity prediction results of trained neural network model

To establish the relationship between the current curve and the wavelength, we drew inspiration from the lifetime fitting methods used in TA spectrum and TRPL analyses^27,41^. The photocurrent curves at various wavelengths and intensities were fitted using a one-phase exponential decay function with a time constant parameter:

$$I={y}_{0}+{A}_{1}{e}^{(-\frac{t}{{t}_{0}})}$$

where *I* is the photocurrent value, *t* is time, A_1_, y_0_, and t_0_ are variables to be fitted. The y_0_ is related to the steady-state photocurrent value, while t_0_ is related to the bending degree of the curve, and A_1_ has little effect on the waveform. Therefore, y_0_ and t_0_ are selected as standards for light wavelength and intensity discrimination. Figure S11 (Supporting Information) illustrates well-fitted photocurrent curves under varying wavelengths of light. Additionally, Figure S12 (Supporting Information) demonstrates that while the waveforms remain consistent at the same wavelength across different light intensities, the curvature of the curve notably increases with rising light intensity, which is reflected in the increase of y_0_ and t_0_ value. We selected a wavelength range from 350 to 750 nm at intervals of 50 nm and conducted calibration with six different light intensities. Corresponding fitting databases of y_0_ and t_0_ values are presented in Figure S13 (Supporting Information), respectively. Both y_0_ and t_0_ exhibit strong correlations with both wavelength and light intensity, suggesting the potential of our dataset for wavelength and light intensity recognition. Subsequently, to enhance our device’s ability to identify the wavelength and intensity of monochromatic light, we directly input the photocurrents of varying wavelengths and intensities into a neural network model for training, as depicted in Fig. 5a. After 100 epochs of training, the model’s loss function converged, reaching nearly 100% accuracy, as depicted in Fig. 5b. When an unknown monochromatic light with unspecified wavelength and intensity illuminated the photodetector, we recorded the current waveform data. To demonstrate the model’s proficiency in discerning the wavelength and intensity of monochromatic light, nine samples were randomly chosen, varying in wavelengths and intensities, for predictive analysis. The results revealed that the model could accurately determine the wavelength of unknown monochromatic light and exhibited an intensity prediction error of less than 0.1%, as shown in Fig. 5c, d. Detailed training procedures and prediction results are provided in Figure S14 (Supporting Information), materials and methods section, and Table S3 (Supporting Information). We anticipate that by expanding the dataset scale, further enhancements in error reduction and resolution can be achieved.

## Discussion

In summary, we have introduced a prototype of a wavelength sensor capable of distinguishing the wavelength and intensity of monochromatic light using only the photocurrent waveform at a single detection point, without the need for additional supporting systems. By adjusting the thickness of 2D perovskite films, we were able to achieve different penetration depths for perovskite absorption with different bandgaps. Furthermore, through the regulation of the charge transport layer against the built-in electric field direction of the 2D perovskite, various photocurrent curve waveforms over time under different wavelengths of light ranging from 350 to 750 nm was obtained. These current waveforms, serving as a database, underwent comprehensive analysis and learning through machine learning model, ultimately achieving precise wavelength recognition with an error rate in light intensity recognition not exceeding 0.1%. Our approach not only provides unique insights into monochromatic light recognition but also offers valuable perspectives and potential applications for the development of future spectrum optoelectronic devices.

## Materials and methods

### Materials

Phenethylammonium iodide (PEAI, >99.5%), methylammonium iodide (MAI, ≥99.5%), lead (II) iodide (PbI_2_, >99.99%), and poly(3-hexylthiophene-2,5-diyl) (P3HT, Mn=10000–100000) were purchased from Xi’an Yuri Solar Co., Ltd. N, N-dimethylformamide (DMF, 99%) and chlorobenzene (CB, 99.8%) solvent were provided by Sigma-Aldrich. Tin (IV) oxide (SnO_2_, 15% in H_2_O colloidal dispersion) was supplied by Alfa Aesar. All the chemicals were used as received without further purification.

### Device fabrication

The device structure for light discrimination is ITO/SnO_2_/perovskite/P3HT/Ag. The ITO glass substrates were ultrasonically cleaned by acetone, ethanol, and deionized water for 15 min, respectively. The SnO_2_ colloidal dispersion was diluted with different volumes of water, and spin-coated on the UV-treated ITO substrates at 5000 rpm for 30 s in ambient. Then the substrates were annealed at 150 °C for 20 min. The substrates were UV-treated for 10 min before transferred to N_2_ glovebox. The 2D perovskite precursor was prepared by dissolving PEAI, MAI, and PbI_2_ at the molar ration of 2:3:4 in DMF, and the precursor concentration is defined by the PbI_2_ content. The perovskite precursor solution was spin-coated on the 100 °C substrate at 5000 rpm for 20 s without ramping. Then, the perovskite films were annealed at 100 °C for 10 min on a hotplate. Afterward, P3HT (10 mg mL^−1^ in chlorobenzene) was spin-coated on the perovskite film at 3000 rpms for 30 s. Finally, 90 nm silver electrodes were thermally evaporated onto the films at a chamber of 10^−4 ^Pa with the deposition rate of 0.5 Å s^−1^.

### Characterizations

XRD patterns were measured by Cu Kα radiation (*λ* = 0.15418 nm, D/Max-III-B-40KV) to analyze the crystallization and crystal orientation of perovskite films. UV-vis spectrum was collected from a UV-vis spectrometer (Shimadzu, UV-3600). The photoluminescence measurements were performed with an Edinburgh FLS 980 instrument, and all the PL measurements were conducted in reflection mode with a 470 nm excitation light from Xenon lamp. The TRPL decays were measured with a time-correlated single photon counting apparatus, together with a 450 nm picosecond laser. To estimate the photoelectric performance, including the current varies with time and responsivity curves, the photodetectors were exposed to the different monochromatic lights from a Xenon lamp together with a monochromatic order sorting filter (Zolix, Omni-*λ* 3009). The corresponding current value was recorded by a semiconductor characterization system (Keithley 4200). The surface and cross-sectional morphologies of films were observed using a field-emission scanning electron microscope (SU8100, Hitachi). The EIS and SCLC characterization were conducted from an electrochemical workstation (Autolab, PGSTAT 302 N). The KPFM spectra were recorded by the MultiMode 8 instrument (Bruker). The response time was recorded by an oscilloscope (Tektronix, MDO3102), and the light was generated from the laser diodes (OSRAM) powered by the function signal generator.

### FDTD simulation

The absorption simulation spectrums of MAPbI_3_ and PEA_2_PbI_4_ were obtained from the Lumerical FDTD software. The simulation models were set as a bulk perovskite of 1.5 μm thickness with periodic boundary condition. The optical constants (refractive index and extinction coefficient) of MAPbI_3_ and PEA_2_PbI_4_ were acquired from other works.

### Neural Network

In this study, the proposed neural network model is developed using the scikit-learn library in Python. A multi-task learning approach was employed to simultaneously recognize wavelength and light intensity. The specifics are outlined as follows:

#### Data Preprocessing

Feature data were standardized using the StandardScaler algorithm from the scikit-learn library, which normalizes the data to have a mean of zero and a standard deviation of one. Wavelength labels were encoded as integers from 0-8 using one-hot encoding. The dataset was also divided into a training set (80%) and a test set (20%) to assess the model’s performance and generalization capability, and to mitigate overfitting.

#### Neural Network Architecture

The neural network comprises three components: an input layer, intermediate hidden layers, and an output layer. The input layer receives the feature data, the intermediate hidden layers extract the most relevant features, compress the input information, and learn the relationships between the features, wavelength, and light intensity. The output layer produces the final predictions. In this model, the output layer is split into two parts: one for predicting wavelength and the other for predicting light intensity.

#### Activation Function

The input layer does not employ an activation function. The activation function for the intermediate hidden layers is Relu, with layers consisting of 512, 256, and 128 neurons, respectively. The output layer for wavelength prediction uses the Softmax activation function, while the output layer for light intensity prediction uses a linear activation function.

#### Optimizer

During training, the Adam optimizer was used, which dynamically adjusts the learning rate for each parameter to improve model parameter updates.

#### Model training

The model was trained over 100 epochs, with a batch size of 32.

## Supplementary information

Supplementary Information for Carrier Dynamic Identification Enables Wavelength and Intensity Sensitivity in Perovskite Photodetectors

## Data Availability

The authors declare that all data supporting the findings of this study are available within the paper and the Supplementary Information, or available from the authors upon request to H.S. (hxsun@suda.edu.cn), W.T. (wtian@suda.edu.cn) or L.L. (lli@suda.edu.cn).
