# Supplementary material for: Carrier dynamic identification enables wavelength and intensity sensitivity in perovskite photodetectors
Source: Light Sci Appl. 2024 Sep 29;13:280. doi: 10.1038/s41377-024-01636-6 (PMC11439907; doi:10.1038/s41377-024-01636-6)
Supplement: Supplementary file 1 — Supplementary Information for Carrier Dynamic Identification Enables Wavelength and Intensity Sensitivity in Perovskite Photodetectors [file 41377_2024_1636_MOESM1_ESM.docx]

Supplementary Information for

**Carrier Dynamic Identification Enables Wavelength and Intensity Sensitivity in Perovskite Photodetectors**

Liangliang Min^1,2^ , Yicheng Zhou^1^, Haoxuan Sun^1🖂^, Linqi Guo^1^, Meng Wang^1^, Fengren Cao^1^, Wei Tian^1🖂^ and Liang Li^1🖂^

^1^School of Physical Science and Technology, Jiangsu Key Laboratory of Frontier Material Physics and Devices, Center for Energy Conversion Materials & Physics (CECMP), Soochow University, Suzhou, 215006, China

^2^College of Physical Science and Technology & Microelectronics Industry Research Institute, Yangzhou University, Yangzhou, 225002, China


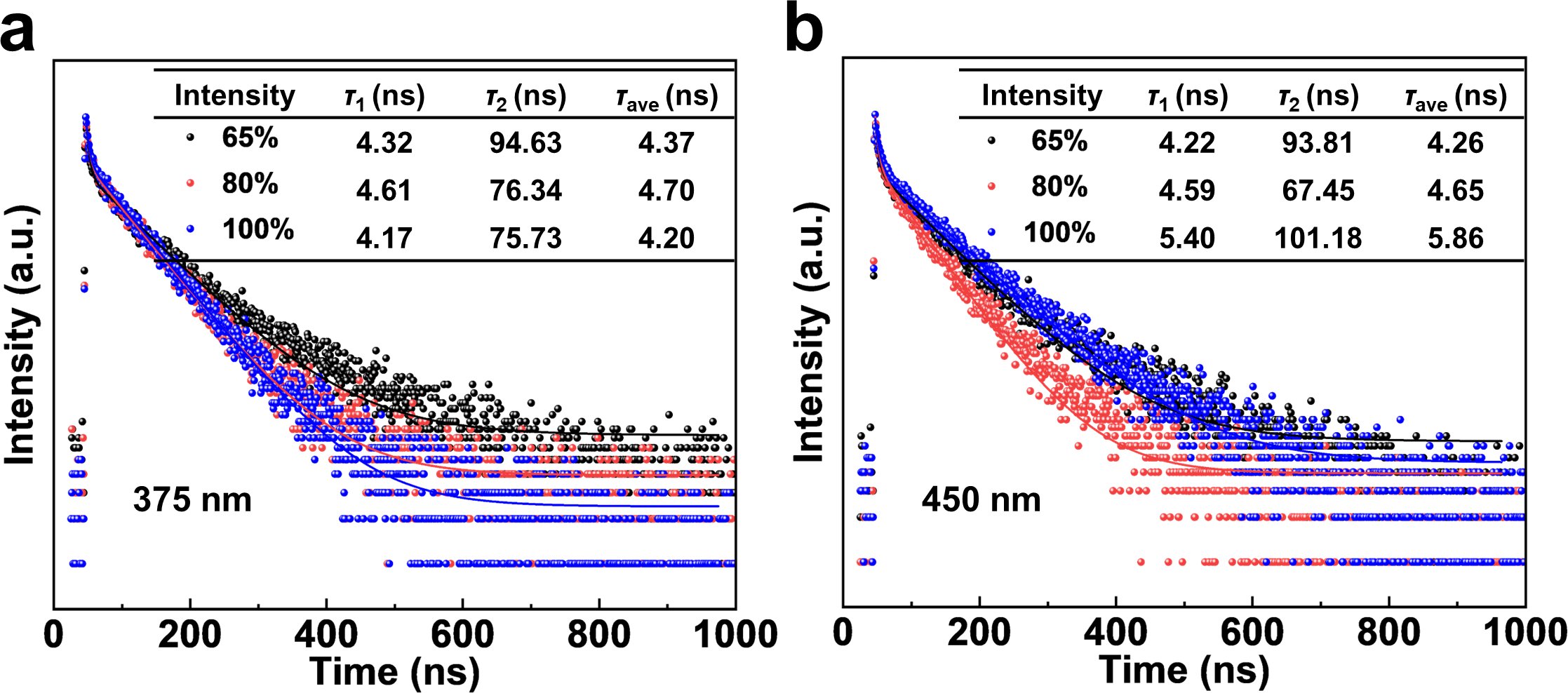


**Figure S1.** The TRPL spectra of 2D perovskites, excited under (a) 375 nm and (b) 450 nm, three light intensities were used for each wavelength.

**
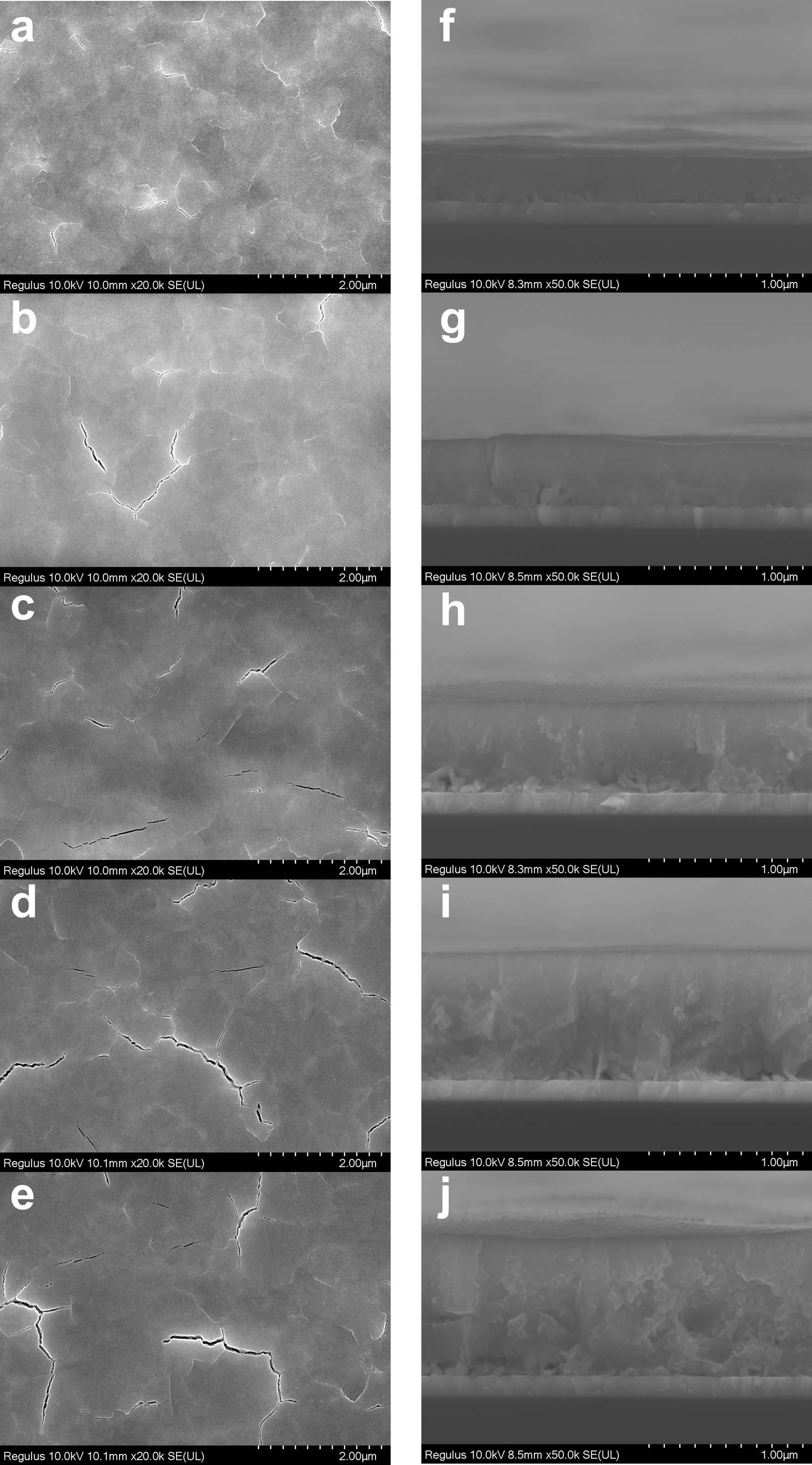
**

**Figure S2.** Top-view and cross-sectional scanning electron microscopy (SEM) images of different thicknesses 2D perovskite films prepared by hot casting method with (a, f) 0.6 M, (b, g) 0.8 M, (c, h) 1.0 M, (d, i) 1.2 M, and (e, j) 1.4 M precursor solution.

**
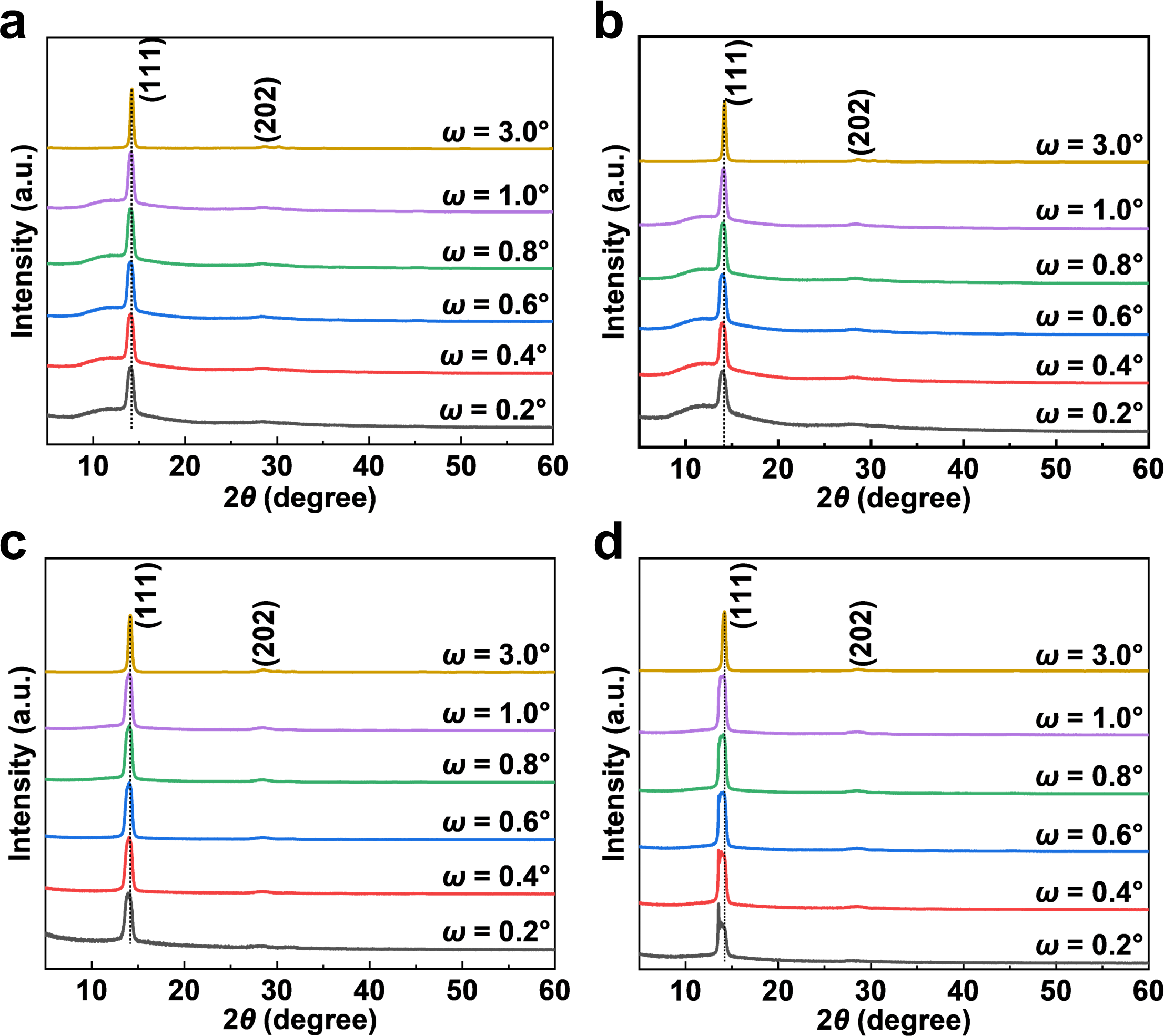
**

**Figure S3.** The GIXRD patterns of different thickness films corresponding to the precursor solution of (a) 0.6 M, (b) 0.8 M, (c) 1.0 M, (d) 1.4 M.


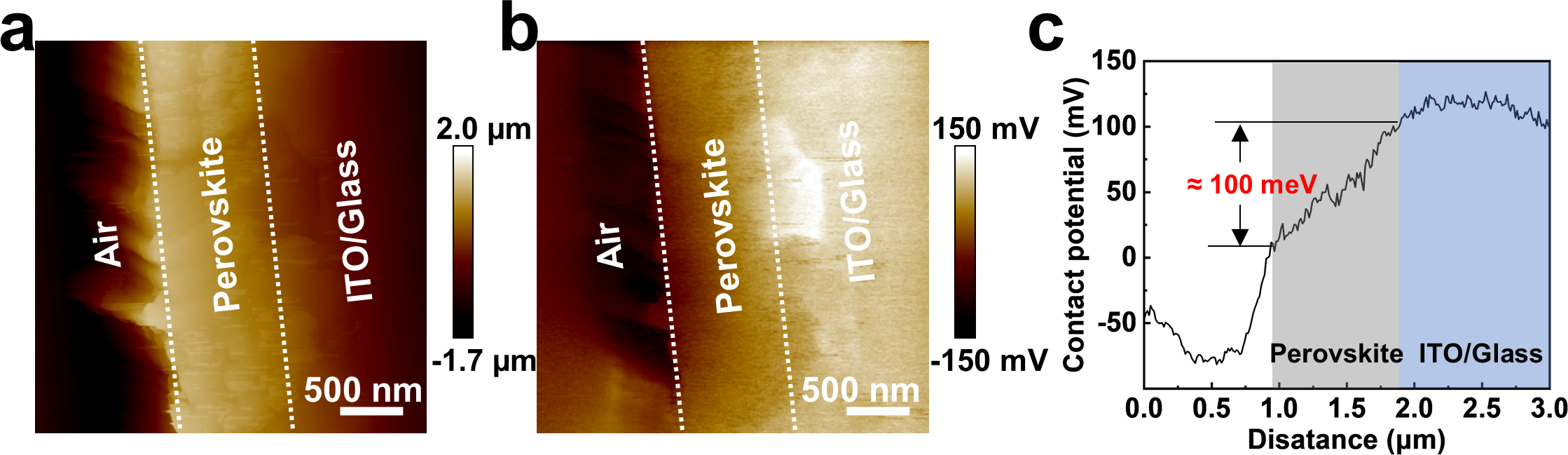


**Figure S4.** (a) Cross-sectional AFM image, (b) KPFM image, and (c) contact potential difference of 2D perovskite on ITO substrate.

**
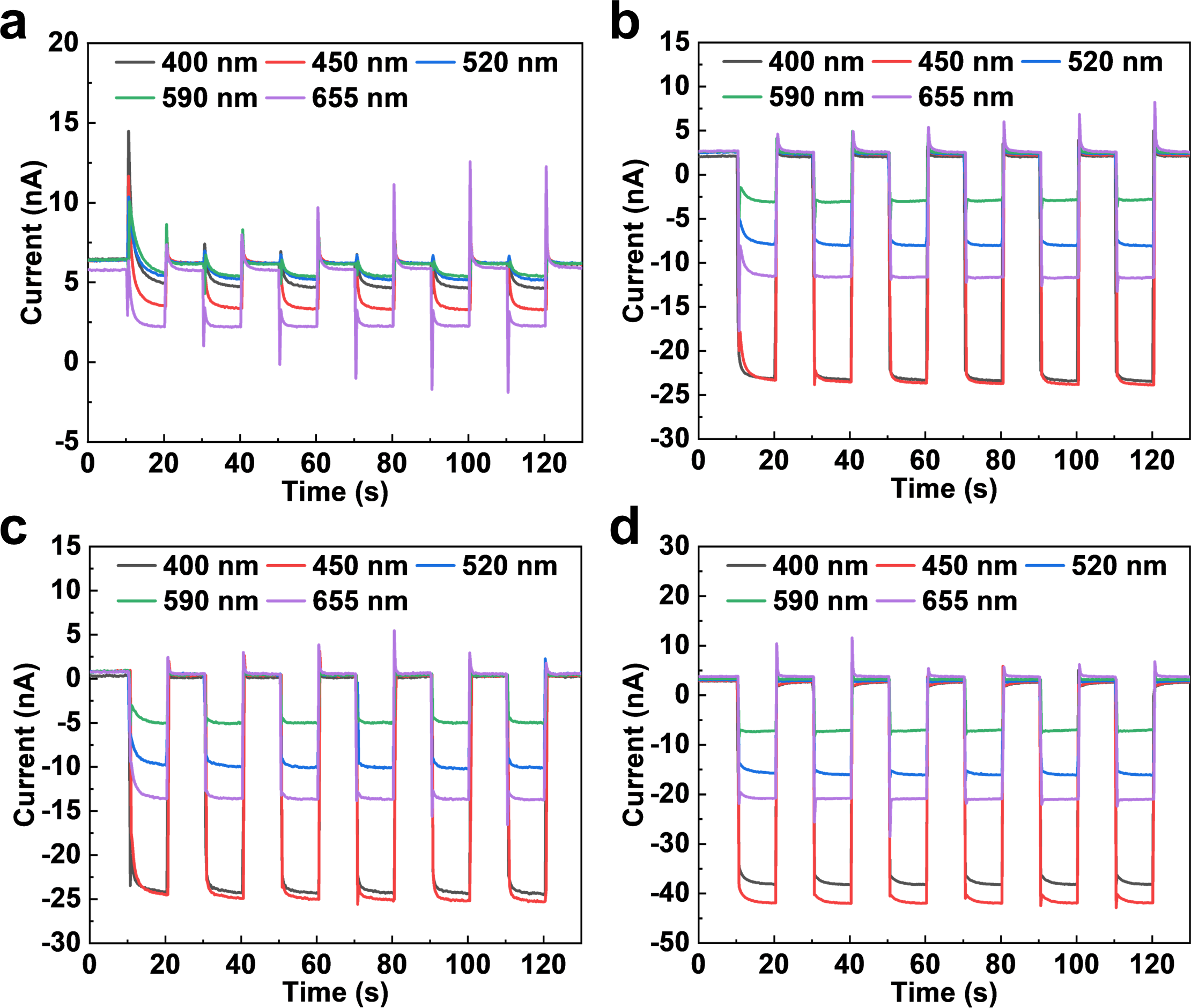
**

**Figure S5.** Photocurrent at different wavelengths without (a) SnO_2_, and the volume ratio of SnO_2_ to water is (b) 1:12, (c) 1:6 and (d) 1:0.

**
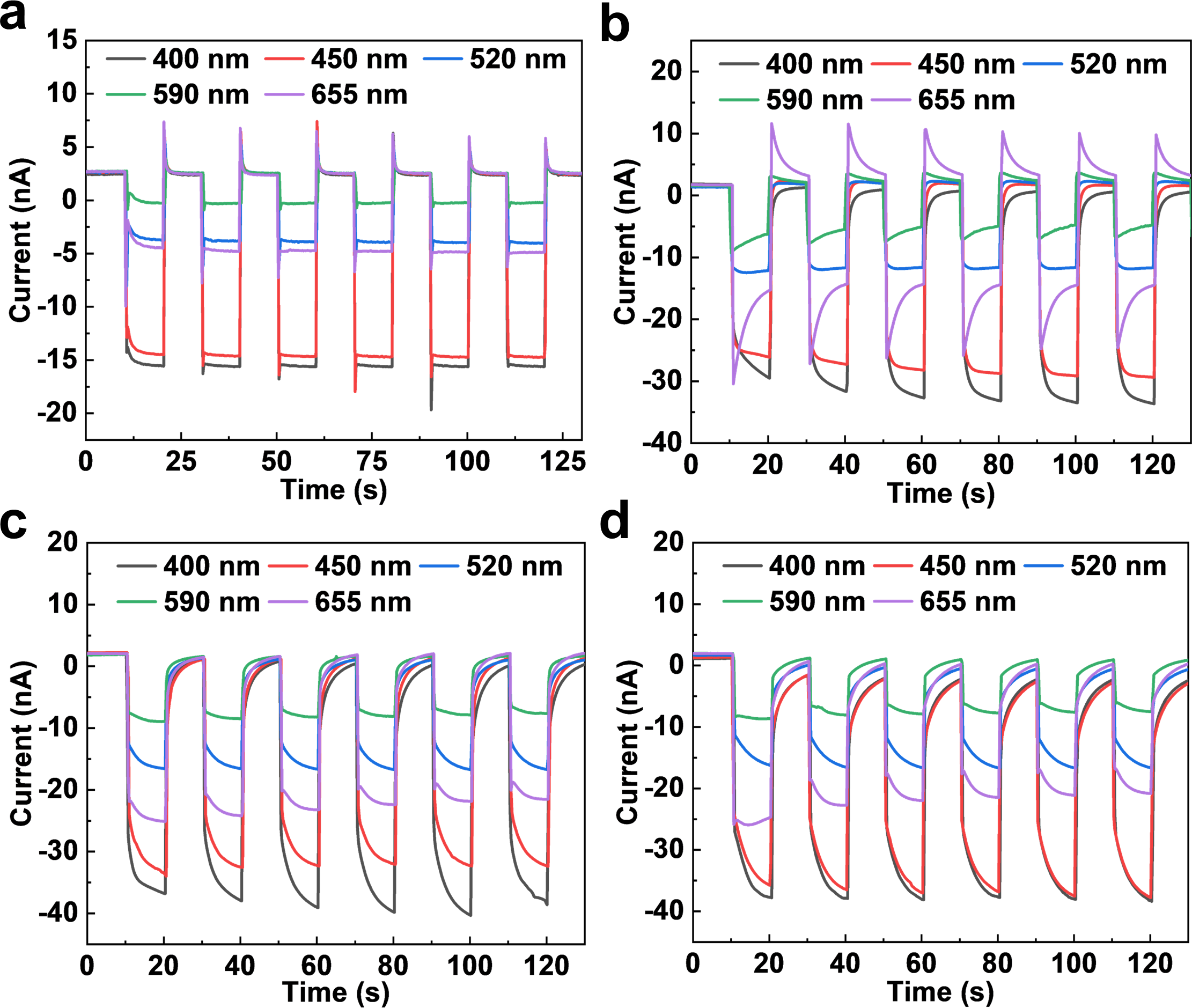
**

**Figure S6.** The photocurrent curves under different wavelengths with different thicknesses HTL prepared by (a) 5 mg mL^-1^, (b) 10 mg mL^-1^, (c) 15 mg mL^-1^, and (d) 20 mg mL^-1^.

**
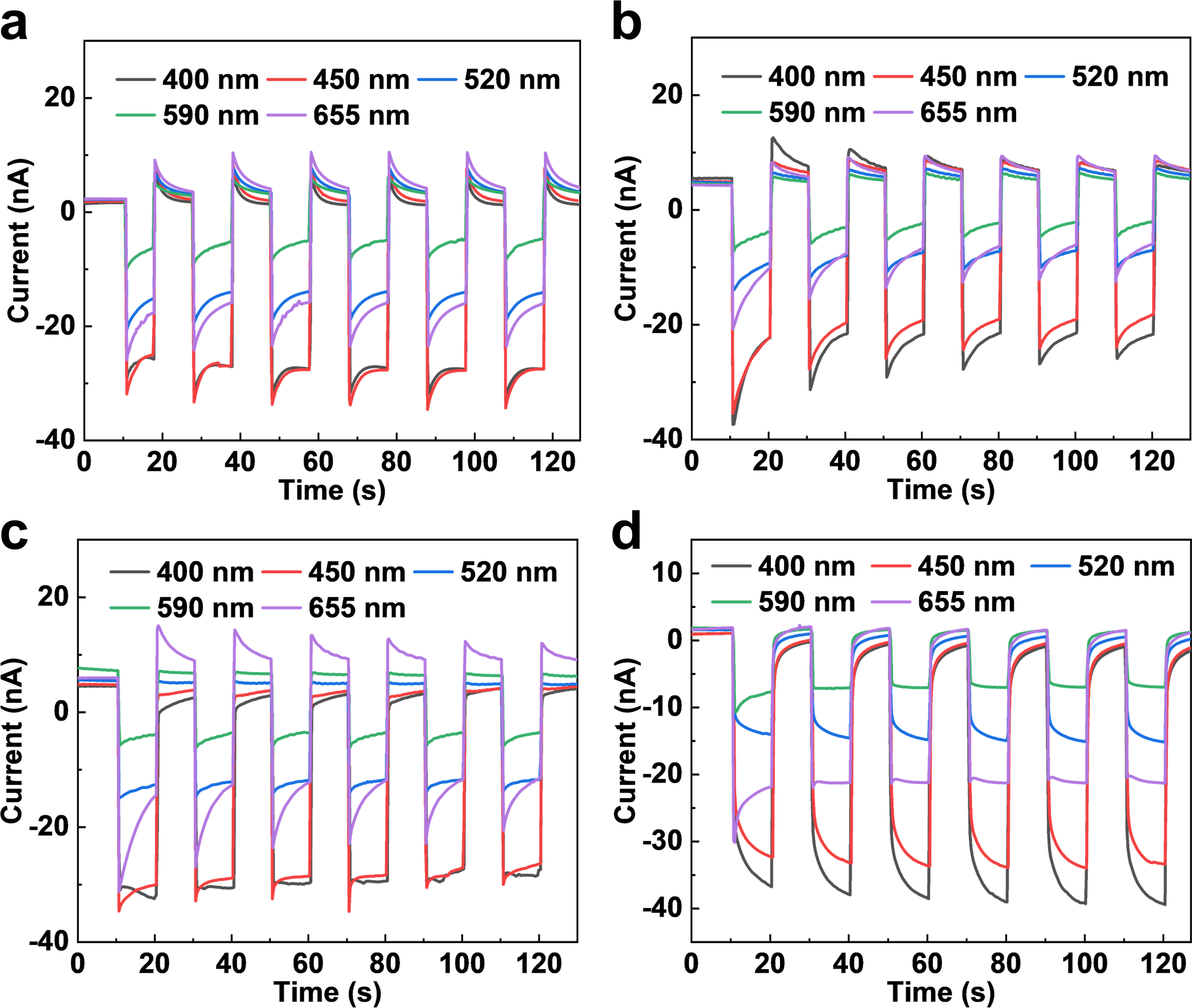
**

**Figure S7.** The photocurrent curves under different wavelengths with (a) 431 nm, (b) 594 nm, (c) 837 nm and (d) 1025 nm thickness perovskite films.


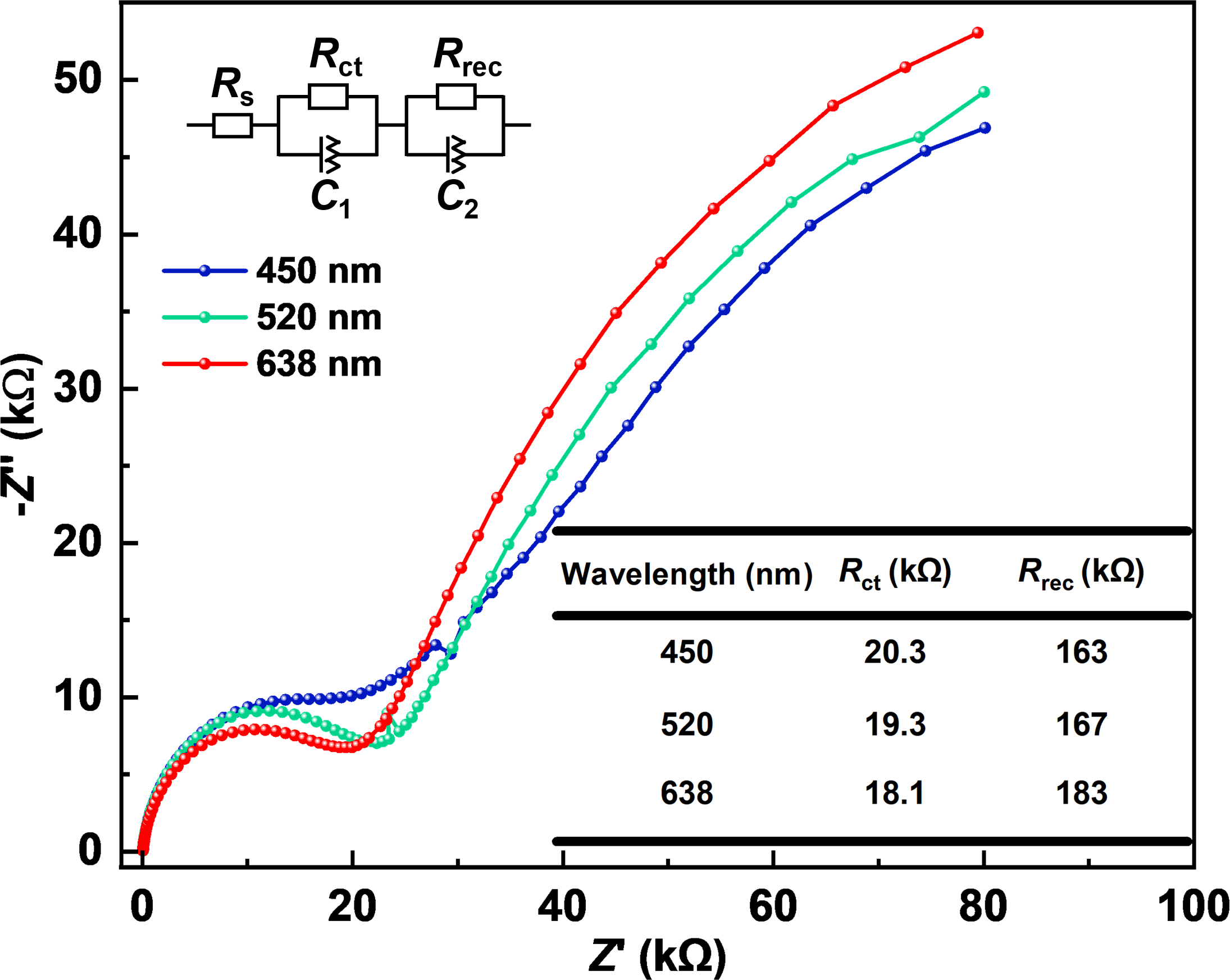


**Figure S8.** The electrochemical impedance spectroscopy of the photodetectors under different monochromatic lights with the same photon numbers.

**
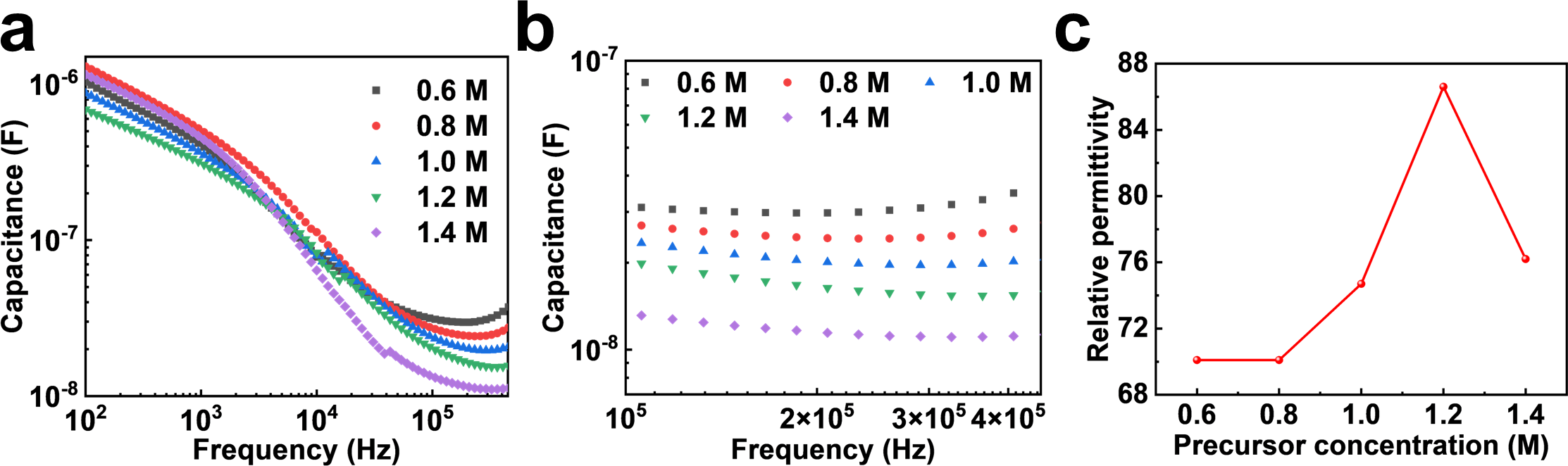
**

**Figure S9.** (a, b) Capacitance as a function of frequency for perovskite films of different thicknesses. (c) calculated relative permittivity.


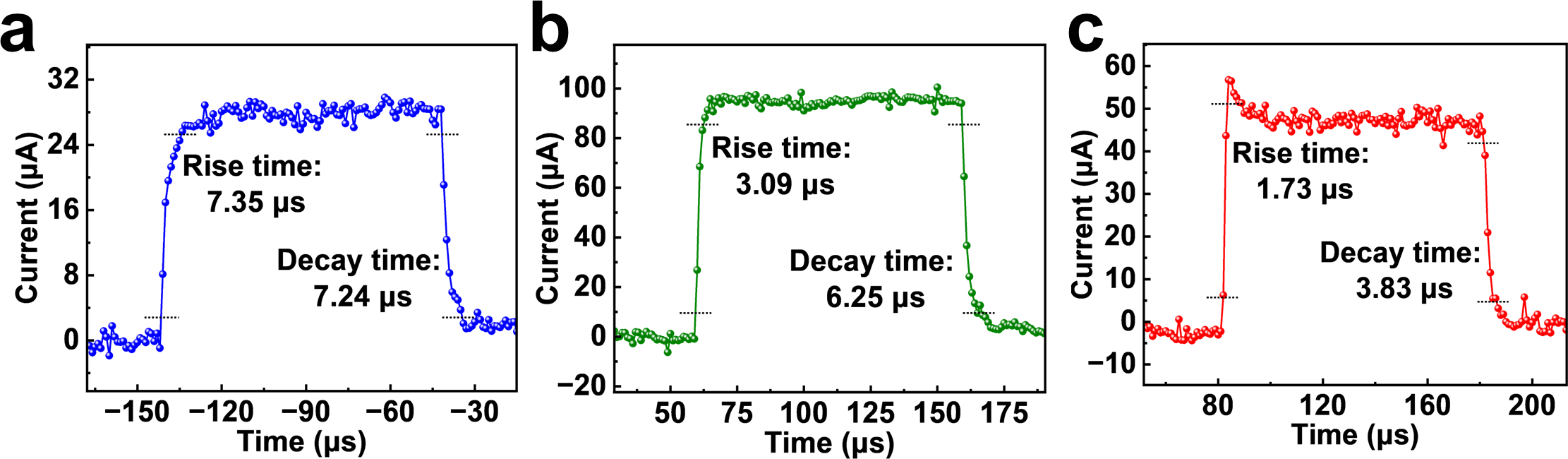


**Figure S10.** The response time under (a) 450 nm, (b) 520 nm, and (c) 638 nm light.

**
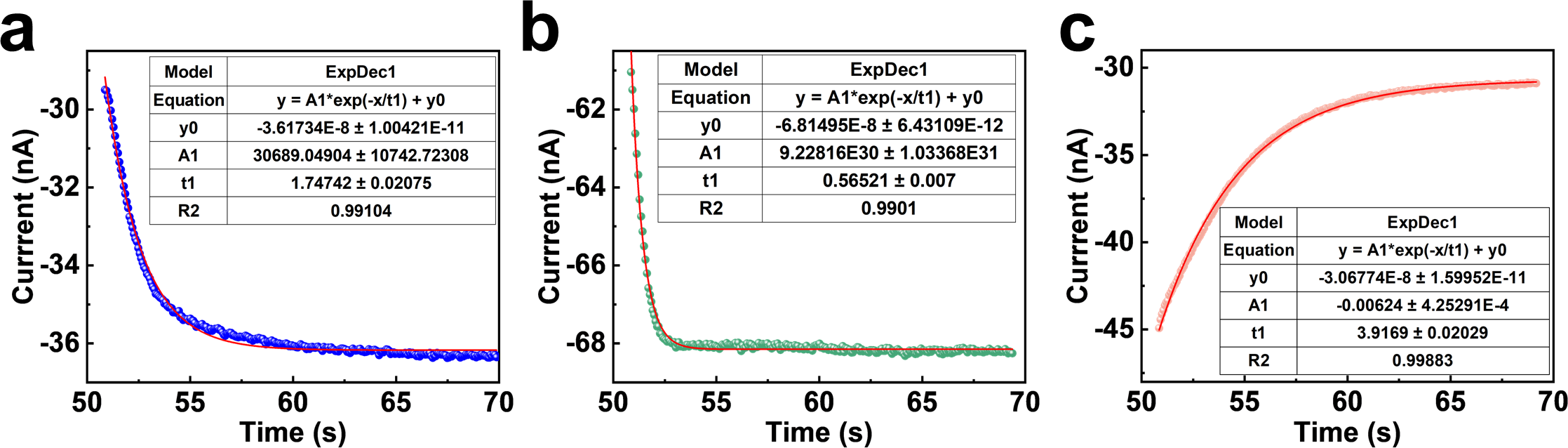
**

**Figure S11.** Photocurrent curves at (a) 400 nm, (b) 500 nm and (c) 650 nm under single exponential fitting.

**
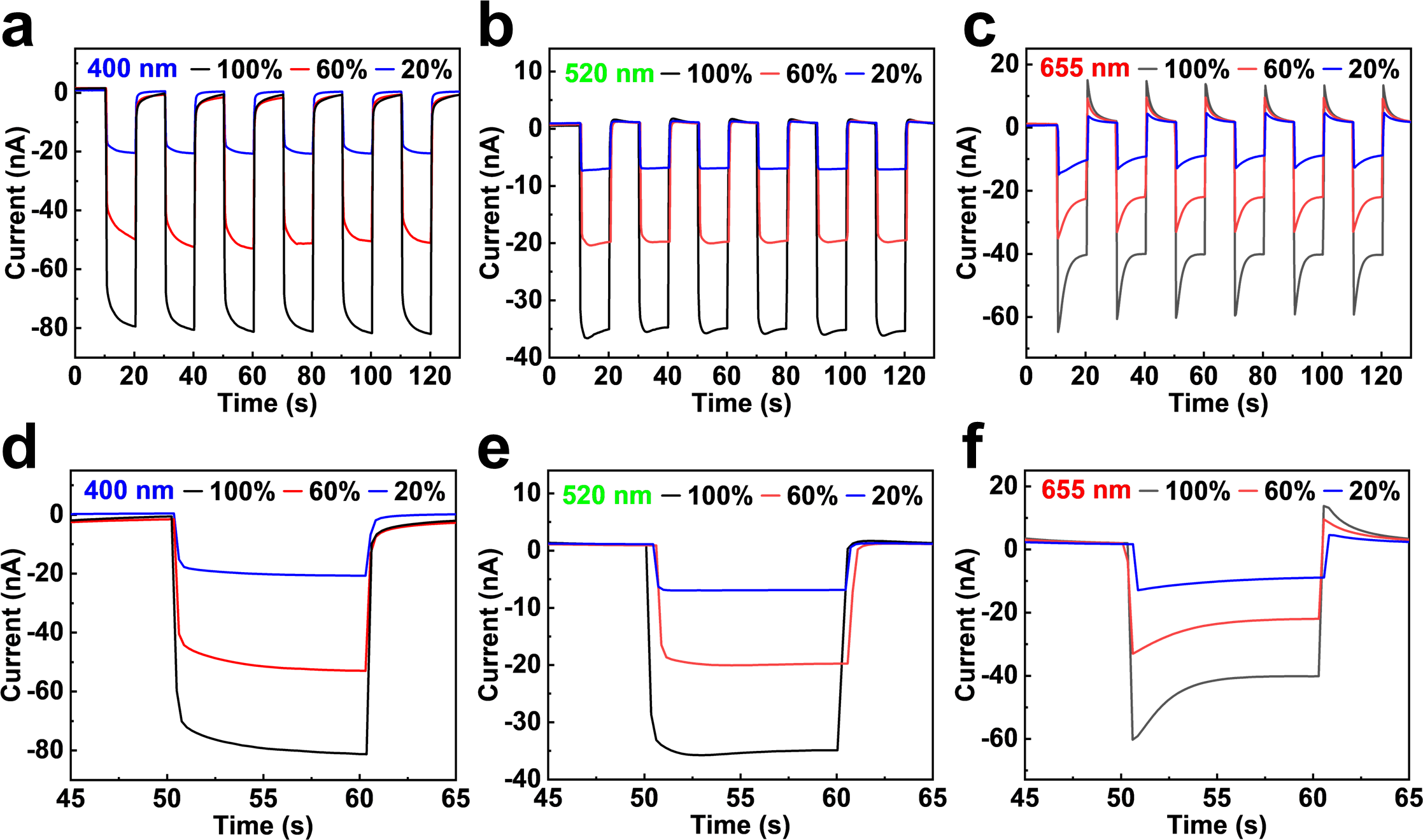
**

**Figure S12.** Photocurrent curves under (a) 400 nm, (b) 520 nm, and (c) 655 nm with three light intensities. (d-f) Enlargement of the photocurrent curves.

**
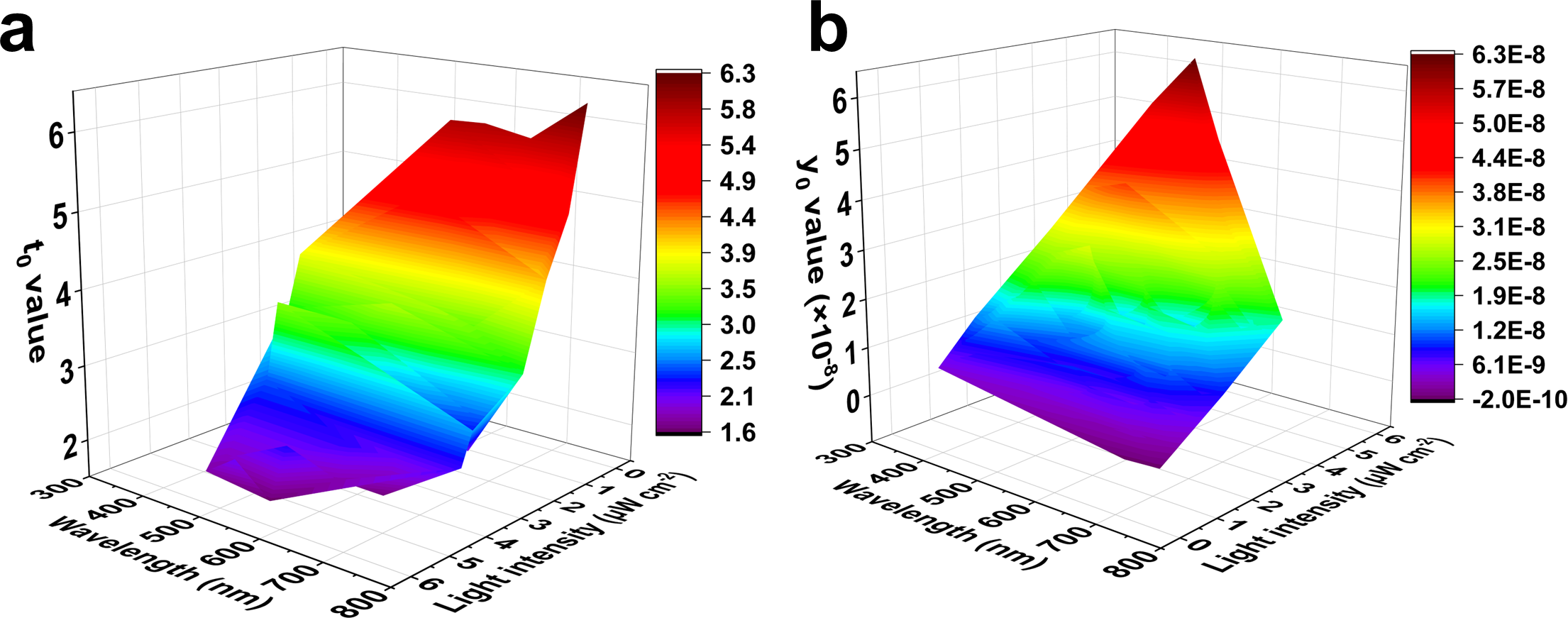
**

**Figure S13.** The fitting databases of (a) t_0_ and (b) y_0_ value as wavelength and light intensity variation.

**
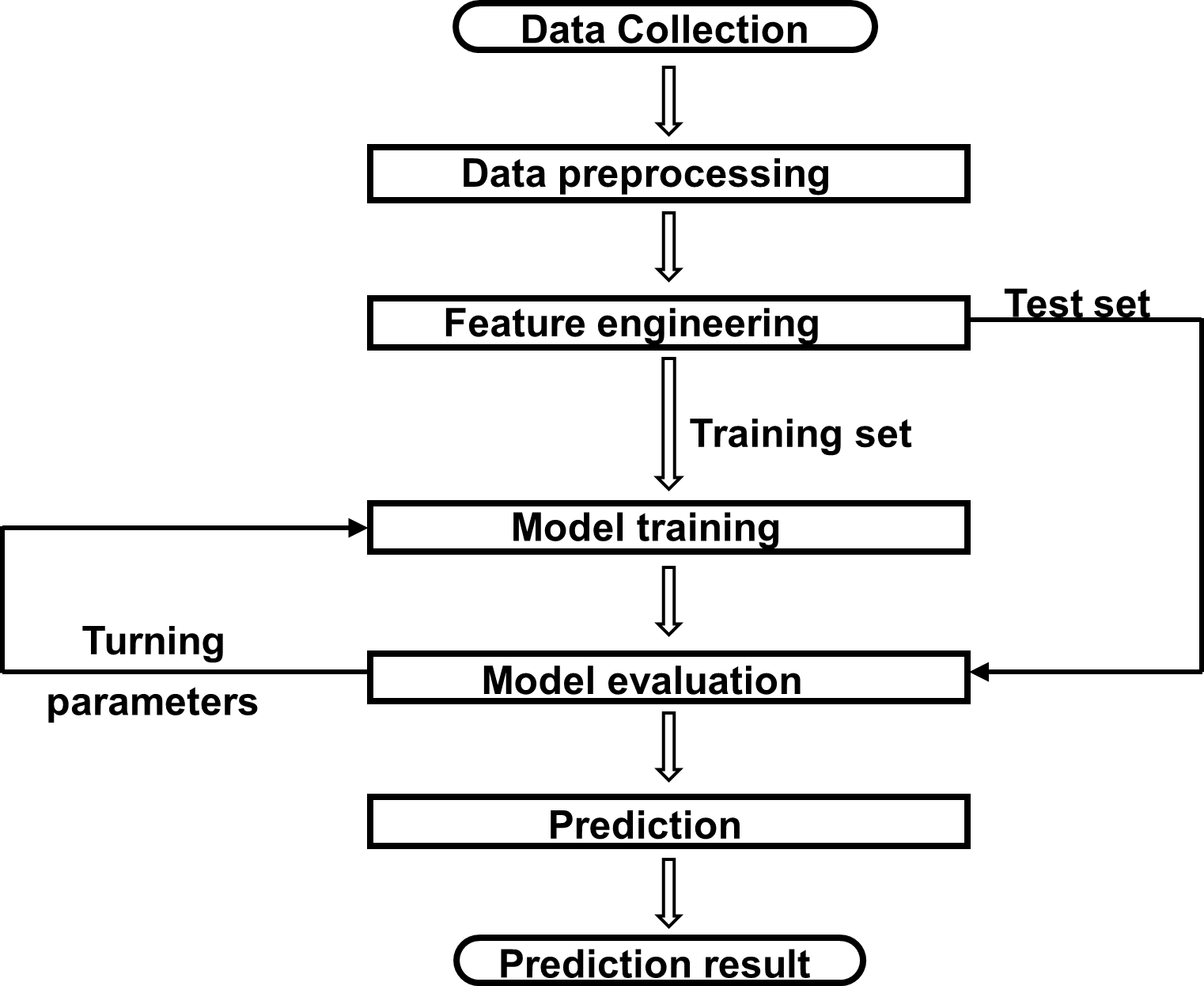
**

**Figure S14.** Flowchart of the neural network model from model training to wavelength and light intensity recognition.

**Table S1.** Impedance fitting results of electrochemical impedance spectroscopy of perovskite films with different thicknesses.

| **Concentration** | ***R*_s_ (Ω)** | ***R*_ct_ (Ω)** | ***R*_rec_ (kΩ)** |
| --- | --- | --- | --- |
| 0.6 M | 20.6 | 609 | 10.8 |
| 0.8 M | 41.4 | 572 | 9.4 |
| 1.0 M | 22.8 | 458 | 29.4 |
| 1.2 M | 21.5 | 441 | 174.0 |
| 1.4 M | 28.9 | 920 | 39.4 |

**Table S2.** The results of TRPL curves for perovskite thin films with varying thicknesses, obtained from the front and back sides, fitted using the double exponential rate law.

| **Front** | **A_1_** | ***τ*_1_ (ns)** | **A_2_** | ***τ*_2_ (ns)** | ***τ*_ave_ (ns)** |
| --- | --- | --- | --- | --- | --- |
| 0.6 M | 1553.5 | 38 | 1.66E+13 | 3.1 | 3.1 |

| 0.8 M | 1.17E+11 | 3.8 | 917.3 | 78 | 3.8 |
| --- | --- | --- | --- | --- | --- |
| 1.0 M | 4.57E+11 | 3.6 | 619.4 | 134.8 | 3.6 |
| 1.2 M | 6.55E+08 | 5.3 | 518.7 | 232.8 | 5.3 |
| 1.4 M | 3.15E+06 | 8.5 | 506.3 | 277 | 9.9 |
| **Back** | **A_1_** | ***τ*_1_ (ns)** | **A_2_** | ***τ*_2_ (ns)** | ***τ*_ave_ (ns)** |
| 0.6 M | 6.54E+07 | 6.5 | 299.4 | 115.6 | 6.5 |
| 0.8 M | 4.02E+06 | 8.6 | 289.8 | 137.7 | 8.7 |
| 1.0 M | 7.71E+05 | 10.5 | 359.8 | 155.4 | 11.5 |
| 1.2 M | 1.59E+05 | 13.5 | 320.4 | 198.7 | 18.8 |
| 1.4 M | 9.83E+04 | 14.9 | 275.4 | 247.5 | 25.2 |

**Table S3.** Results and errors of the neural network trained to predict the wavelength and intensity of unknown monochromatic light.

| ***λ*_true_ (nm)** | ***λ*_predicted_ (nm)** | ***I*_true_ (nW cm^-2^)** | ***I*_predicted_ (nW cm^-2^)** | **Error (%)** |
| --- | --- | --- | --- | --- |
| 350 | 350 | 1162.666668 | 1162.666500 | 0.0146 |
| 400 | 400 | 1321.48148 | 1321.480800 | 0.0516 |
| 450 | 450 | 2254.545456 | 2254.543500 | 0.0871 |
| 500 | 500 | 3369.630354 | 3369.638400 | -0.0239 |
| 550 | 550 | 2195.524148 | 2195.521700 | 0.0112 |
| 600 | 600 | 1810.256412 | 1810.257100 | -0.0038 |
| 650 | 650 | 1653.333332 | 1653.332500 | 0.0049 |
| 700 | 700 | 1060.869564 | 1060.869600 | -0.0038 |
| 750 | 750 | 1512.236288 | 1512.237000 | -0.0047 |
